# Supplementary material for: NaF-PET Imaging of Atherosclerosis Burden
Source: J Imaging. 2023 Jan 30;9(2):31. doi: 10.3390/jimaging9020031 (PMC9966512; doi:10.3390/jimaging9020031)
Supplement: Supplementary file 1 [file jimaging-09-00031-s001.zip › jimaging-2012405-supplementary.pdf]

Journal of Imaging

Supplementary Material

STATE-OF-THE-ART REVIEW

NaF-PET Imaging of Atherosclerosis Burden

Poul F. Høilund-Carlsen, Reza Piri, Oke Gerke, Michael Sturek, Thomas J. Werner, Mona-Elisabeth Revheim, Abass Alavi

Contents

Search Strings for Literature Retrieval 2

Table S1: Apr 2020 – Dec 2022 Studies on Disease Mechanisms and Targeting 3

Table S2: Apr 2020 – Dec 2022 Studies on Early Detection and Prevalence of NaF Uptake in the Heart and Major Arteries 5

Table S3: Apr 2020 – Dec 2022 Studies on NaF Uptake in Vulnerable, High-Risk and Ruptured Plaque 7

Table S4: Apr 2020 – Dec 2022 Studies on Association Between Arterial NaF Uptake and Risk Factors 9

Table S5: Apr 2020 – Dec 2022 Studies on NaF Uptake and Disease Progression or 'Prediction' of Events 12

Table S6: Apr 2020 – Dec 2022 Studies on Anti-Atherosclerotic Intervention Evaluated by NaF PET/CT 15

Discussion of Minor Findings 17

References 19

Full search strategies for Pubmed/Medline, Web of Science, and Embase

(((((Positron emission tomography[Title/Abstract]) OR (PET [Title/Abstract]) OR (PET/CT[Title/Abstract]) OR (PET/MRI[Title/Abstract]))

AND

(((Sodium Fluoride[Title/Abstract]) OR (NaF[Title/Abstract]) OR (18 NAF[Title/Abstract]) OR (NaF-PET[Title/Abstract]) OR (NAF F18[Title/Abstract]) OR (Sodium fluoride F18[Title/Abstract]) OR (NaF-PET-CT[Title/Abstract]) OR (18F-NaF[Title/Abstract]) OR (18-F-NaF[Title/Abstract]) OR (18-F NaF[Title/Abstract]) OR (F-18 NaF[Title/Abstract])))

AND

(((Atherosclerosis[Title/Abstract]) OR (Artery[Title/Abstract]) OR (stenosis[Title/Abstract]) OR (narrowing[Title/Abstract]) OR (atherosclerotic[Title/Abstract]) OR (Plaque[Title/Abstract]) OR Atheroma[Title/Abstract]) OR (artery disease[Title/Abstract]) OR (cardiovascular[Title/Abstract]) OR (heart[Title/Abstract]) OR (arteries[Title/Abstract]) OR (cardiac[Title/Abstract])))) NOT (Review[Publication Type]) AND (2000:2022[pdat])

Embase

1. PET.mp. or exp positron emission tomography/
2. fluorine 18/ or positron emission tomography-computed tomography/ or fluoride sodium/ or fluoride/ or NaF.mp.
3. Atherosclerosis.mp. or carotid atherosclerosis/ or brain atherosclerosis/ or exp atherosclerosis/ or aortic atherosclerosis/ or coronary artery atherosclerosis/ or experimental atherosclerosis/
4. 1 and 2 and 3
5. limit 4 to yr="2000 -Current"

Scopus

(positron AND emission AND tomography OR pet OR pet/ct OR pet/mri) AND (sodium AND fluoride OR naf OR 18 naf OR naf-pet OR naf AND f18 OR sodium AND fluoride AND f18 OR naf-pet-ct OR 18f-naf OR 18-f-naf OR 18-f AND naf OR f-18 AND naf) AND (atherosclerosis OR artery OR stenosis OR narrowing OR atherosclerotic OR plaque OR atheroma OR artery AND disease OR cardiovascular OR heart OR arteries OR cardiac)

Cochrane library

"Positron emission tomography" OR "PET" OR "PET/CT" OR "PET/MRI" in Title Abstract Keyword AND “sodium fluoride“ OR “naf“ OR “18 naf“ OR “naf pet“ OR “naf pet ct“ OR “18f naf“ OR “18 f naf“ OR “18 f naf“ OR “f 18 naf“ in Title Abstract Keyword AND “atherosclerosis“ OR “artery“ OR “stenosis“ OR “narrowing“ OR “atherosclerotic“ OR “plaque“ OR “atheroma“ OR “artery disease“ OR “cardiovascular“ OR “heart“ OR “arteries“ OR “cardiac“

Table S1. Apr 2020 – Mar 2022 Studies on Disease Mechanisms and Targeting.

First author, time [Ref. #] Site	Study subjects, n (females) Age in years Mean±SD or range	Material	Tracer	Arterial segment	Purpose	Quantification	Main findings	Comment
Zhuang et al. 2021 Feb [5] Luzhou, China.	19 male ApoE-/- rats and 3 normal ones on normal diet. One normal male rat for MRI defined aortic and pulmonary VOIs.	ApoE-/- rats fed on high-fat diet from 13 weeks of age, with increased weight and cholesterol levels with time.	NaF & FDG	Aortic arch and proximal pulmonary artery segments	Longitudinal in vivo study to monitor inflammation and calcification during formation of atherosclerotic plaques in ApoE-/- rats by FDG and NaF PET/CT.	PET/CT at baseline (12 weeks) and at 27 and 46 weeks of age. SUVmax and SUVmean but no actual values, only statements of higher or lower followed by a p-value.	FDG accumulated in the aortic arch, while NaF accumulated in one or both pulmonary arteries. FDG uptake was significantly higher in the target group at 46 weeks than in controls, while NaF uptake was significantly higher at both 27 and 46 weeks of age. Thus, the two tracers were not present in the same arteries and FDG did not appear earlier than NaF. HIF-1α staining indicated that the aortic arch with high FDG uptake had higher expression of hypoxia than the pulmonary arteries with no FDG uptake. NaF correlated with microcalcifications in lesions; but low spatial PET resolution meant that exact tracer location could not be determined.	Pulmonary atherosclerosis has been reported in mice; this appears to be the first study describing this finding in rats.
Nogales et al. 2021 Jul 22 [6] Madrid, Spain & Aarhus, Denmark.	4 female PCSK9j^{D374Y} Minipigs.	Transgenic Yucutan minipigs fed on high-fat diet for 2.5 years to develop atherosclerosis.	NaF	Abdominal aorta, iliac ateries, coronary arteries.	Validation of the specificity of NaF PET for plaque calcifications in atherosclerotic minipigs.	TBRmax and TBRmean derived from SUV values divided by blood pool activity in abdominal vena cava or left ventricle.	In vivo imaging compared to rescans after surgical extraction of heart, aorta and iliac arteries and to histological plaque findings and autoradiography. Arterial NaF uptake co-localized moderately with CT calcification. Autoradiography showed specific accumulation of NaF in plaque calcifications in all examined arteries. However, to what degree NaF is taken up by microcalcifications and possibly also thrombotic material or small calcifications in thrombi in these human-like early plaques could not be determined.	Authors found "NaF PET less accurate than CT for detecting small calcifications" disregarding that microcalcifications < 50 µm can be observed with NaF-PET, whereas CT detection requires size 200-500.
Omarjee et al. 2020 Oct 27 [7] Rennes/ Angers, France.	23 (12) PXE pts 47.2±14.1 y & 23 (12) healthy controls 46.1±13.2 y	PXE vs healthy controls.	NaF & FDG	Carotid, asc. + arch, desc. + abdominal aorta, iliac, femoral. Popliteal as reference.	Skin/artery inflammation and calcfication in PXE pts.	Arterial TBRmax (and skin SUVmax), carotid-femoral pulse wave velocity (PWV) and CT calcifiation.	Significant FDG uptake in diseased skin areas contrary to normal regions, and exclusively in proximal aorta contrary to popliteal arteries. No correlation btw. FDG uptake and PWV in aortic wall. Significant 18F-NaF uptake in diseased skin regions and in proximal aorta and femoral arteries. NaF wall uptake correlated with calcium score in tfemoral arteries, and aortic wall PWV. Aortic wall NaF uptake associated with diastolic blood pressure. No significant correlation btw. FDG and NaF uptake in any of the artery walls. Thus: inflammation and calcification were not correlated. PXE more closely resembles chronic disease model of ectopic calcification than inflammatory condition.	
Altonen et al. 2022 Jan 3 [8] Turku, Finland.	32 (13) 55 (37-83) y	Pts on dialysis, 12 with diabetes, 19 with CVD, 6 with prior MI; various medications.	NaF	NaF uptake in lumbar spine and iliac crest vs. CT calcification score in coronary arteries and aorta.	To evaluate the possible relationship between arterial calcification and bone metabolism.	Patlak analysis to estimate plasma clearance of NaF into bone. Calcification scores by CT of 0, 1,2,3 in the aorta (AAC) and coronary arteries (CAC), the latter	High prevalence of arterial calcification and 59% had verified CVD. Both CAC and AAC were significantly higher in patients with verified CVD. Only 22% had low turnover bone disease. Weak association between NaF activity, reflecting bone turnover measured in the lumbar spine and CAC, and between parathyroid hormone and CAC. Weak association between erosion surfaces and AAC. No significant association between calcification score and any other parameters The results highlight the complexity when evaluating the link between bone remodeling and vascular calcification in patients with multiple comorbidities and extensive atherosclerosis.	Circumstantial study in that NaF uptake was not measured in heart or large arteries, but in the lumbar vertebrae and compared a CT-based calcification in coronary arteries and aorta. One of few studies trying to shed light on a potential link between

<table>
<tr><th>First author, time [Ref. #] Site</th><th>Study subjects, n (females) Age in years Mean±SD or range</th><th>Material</th><th>Tracer</th><th>Arterial segment</th><th>Purpose</th><th>Quantification</th><th>Main findings</th><th>Comment</th></tr>
<tr><td></td><td></td><td></td><td></td><td></td><td></td><td>by Agatston grades 0, 1-100, 101-399, ≥400.</td><td></td><td>atherosclerosis and bone metabolism.</td></tr>
<tr><td>Wen et al. 2022 Apr 12 [9] Beijing, China and Vienna, Austria</td><td>100 (24) 64 (57-68) Y</td><td>Pts with multivessel CAD</td><td>NaF</td><td>Proximal LAD, LCX, RCA vs. ascending aorta and arch.</td><td>Association btw coronary and aortic NaF uptake and pro-atherosclerotic factors in pts with multivessel CAD.</td><td>Average of TBRmean in the three coronaries vs. aortic parts. PCAT by CT (-190 to -30 HU).</td><td>Nice positive correlation btw coronary and aortic NaF uptake. Coronary NaF uptake was significantly and positively correlated with PCAT.</td><td></td></tr>
<tr><td>Raynor et al. Oct 2022 [10] Philadelphia, USA, Odense, Denmark, Oslo, Norway</td><td>(1) 33 men 70±6.6 y (2) 33 (20) 64±5 y (3) 33 (17) 59±5.2 y</td><td>(1) Men with prostate cancer (PC). (2) Pts with angina pectoris (AP). (3) Healthy control men and women (HC).</td><td>NaF</td><td>Proximal coronary arteries, ascending aorta, arch, descending (thoracic) aorta.</td><td>Comparison of NaF uptake in the coronary arteries, and aorta in prostate cancer patients, patients with angina pectoris and healthy controls.</td><td>Global SUVmean in the arterial segments divided by average NaF activity in section of abdominal fat = TBRmean.</td><td><table>
<tr><th>Vessel</th><th>HC</th><th>AP</th><th>PC</th><th>HC vs AP</th><th>HC vs PC</th></tr>
<tr><td>Coron.</td><td>4.2 ± 1.4</td><td>4.9 ± 3.4</td><td>5.3 ± 2.1</td><td>p=0.21</td><td>p=0.15</td></tr>
<tr><td>Asc.</td><td>5.4 ± 1.6</td><td>6.7 ± 3.5</td><td>6.6 ± 2.4</td><td>p=0.03</td><td>p=0.04</td></tr>
<tr><td>Arch</td><td>5.7 ± 1.9</td><td>6.9 ± 3.0</td><td>6.8 ± 2.6</td><td>p=0.04</td><td>p=0.03</td></tr>
<tr><td>Desc.</td><td>6.1 ± 1.7</td><td>7.2 ± 3.9</td><td>7.5 ± 3.0</td><td>P=0.07</td><td>p=0.04</td></tr>
</table>Thus lowest uptake in coronary arteries and tendency for higher uptake in all segments in AP and PC, with at similarly high uptake in PC compared to AP patients.</td><td></td></tr>
</table>

ApoE-/- = apolipoprotein E knockout; CAD = coronary artery disease; CT = computed tomography; CV = cardiovascular; CVD = cardiovascular disease; FDG = 18F-fluorodeoxyglucose; HU = Hounsfield units; MI = myocardial infarction; NaF = 18F-sodium fluoride; PCAT = peri-coronary adipose tissue; pts = patients; PXE = pseudoxanthoma elasticum; SUV = standardized uptake value; TBR = target-to-background ratio; VOI = voxel of interest; Y = years.

Table S2. Apr 2020 – Mar 2022 Studies on Early Detection and Prevalence of NaF Uptake in the Heart and Major Arteries.

First author, time (Ref. #) Site	Patients, n (females) Age in years Mean±SD or range	Material	Tracer	Arterial segment	Purpose	Quantification	Main findings	Comment
Zhang et al. Apr 2020 [11] Philadelphia, USA & Odense, Denmark.	15 (0) healthy controls, 45±8 y and 15 (6) chest pain pts 56±11 y	Healthy controls vs. chest pain pts with 10-year risk for fatal CV disease ≥ 1% by the SCORE tool.	NaF	Manual ROIs around main, right, and left pulmonary artery on axial slices.	Do at-risk patients have increase pulmonary artery NaF uptake compared to healthy controls?	Global SUVmean and SUVmax and TBRmean	For global SUVmean (0.79 vs. 0.58), global TBRmean (1.15 vs. 0.93), and global SUVmax (1.78 vs. 1.60), NaF uptake was significantly higher in the at-risk patients compared to the controls (all P<0.05).	First study to investigate the role of NaF-PET/CT for assessment of atherosclerosis calcification in the pulmonary artery.
Gutierrez-Cardo et al. May 2020 [12] Málaga, Spain.	18 (13) 47±13 y	PXE pts.	NaF	Carotids, 4 aortic sections, iliac, femoral, popliteal arteries.	Quantification of arterial and skin depositions of NaF.	TBRmax and TBRmean. CT calcium deposits by score, volume, mass.	Higher vascular calcification in popliteal, femoral, and aortic arch vessels, highest in the aorta and femoral arteries. Highest skin uptake in neck and axillae. No significant association between NaF deposits in arteries and skin or between NaF uptake and global Phenodex score. In contrast, Phenodex score was significantly associated with averaged vascular CT calcium score (p < 0.01). In the neck, pts with higher skin Phenodex scores exhibited higher radiotracer uptake.	
Seraj al. Jun 2020 [13] Philadelphia, USA & Odense, Denmark.	18 RA patients (4), 56.0±11.7 y and 18 healthy controls (4) 55.8±11.9 y	Controls were matched to patients by sex and age (±4 years).	NaF and FDG	Abdominal aorta.	Can NaF-PET/CT or FDG-PET/CT detect abdominal aortic molecular calcification and inflammation in patients with RA?	TBRmean CT calcium volume score.	Average NaF TBRmean among RA patients was significantly greater than among healthy controls (median 1.61; IQR 1.49–1.88 and median 1.40; IQR 1.23–1.52, P=0.002). Average CT calcium volume score among RA patients was also significantly greater (median 1.96 cm3; IQR 0.57–5.48 and median 0.004 cm3; IQR 0.04–0.05, P<0.001). No signif. difference between average FDG TBRmean scores in RA patients compared to healthy controls (median 1.29; IQR 1.13–1.52 and median 1.29; IQR 1.13–1.52, respectively, P=0.98).	Post hoc analysis.
Asadollahi et al. Dec 2020 [14] Philadelphia, USA & Odense, Denmark.	5 (4) 61.8±6.6 y, with PAD vs. 5 (4) without PAD 60±7.2 y	Pts with PAD matched to pts without PAD based on age and gender from the at-risk cardiovascular risk profile group of the CAMONA trial.	NaF	Coronary artery (CA), carotid (CR), ascen-ding (AS), arch (AR), descending aorta (DA), abdominal aorta (AA).	Comparison of atherosclerotic burden in non-lower extremity arteries in patients with and without PAD using NaF-PET/CT.	Manual segmentation. Average SUVmean (aSUVmean) was calculated for each segment.	Total aSUVmean was higher in the PAD group compared to the non-PAD group (6.54±0.9 vs. 5.03±0.45, P=0.043). Comparison revealed higher NaF uptake in CR, AS, AR, and DA in the PAD group compared to the non-PAD group (0.93±0.25 vs. 0.54±0.14, P=0.01; 1.28±0.20 vs. 0.86±1.19, P<0.01; 1.18±0.17 vs. 0.90±0.19, P=0.03; 1.32±0.24 vs. 0.91±0.15, P=0.01). The NaF uptake in CA and AA was similar in the two groups (0.77±0.04 vs. 0.71±0.05, P=0.11; 1.07±0.28 vs. 1.12±0.30, P=0.82). Individuals with PAD had higher atherosclerotic burden in the carotid	

First author, time (Ref. #) Site	Patients, n (females) Age in years Mean±SD or range	Material	Tracer	Arterial segment	Purpose	Quantification	Main findings	Comment
							arteries and thoracic aorta compared to non-PAD subjects.	
Bhattaru et al. Feb 2021 [15] Philadelphia, USA & Odense, Denmark.	68 (35) healthy (42±13.5 y vs. 40 (22) at-risk 55±11.9 y	Healthy controls vs. patients at-risk for cardiovascular disease.	NaF	Coronary artery (CA), asc., arch, desc. and abdominal aorta (AS, AA, DA, AA); common & ext. iliac artery (CIA &EIA), femoral & popliteal artery (FA & PA).	To quantify the heterogeneity of atherosclerosis in upper and lower limb vessels using NaF-PET/CT and to compare calcification in coronary arteries to peripheral arteries.	Average SUVmean (aSUVmean) was calculated for each arterial segment.	CA aSUVmean in the at-risk group was higher than in the healthy control group (0.74±0.04 vs. 0.67±0.04, P=0.03). Furthermore, the NaF uptake in CA was lower than in AS, AR, DA, AA, CIA, EIA, FA, and PA in both healthy (all P≤0.0001) and at-risk (all P≤0.0001). Higher NaF uptake in non-cardiac arteries in both healthy controls and patients at-risk suggests CA calcification is a late manifestation of atherosclerosis.	This differential expression of atherosclerosis is likely due to interaction of hemodynamic parameters specific to the vascular bed and systemic factors related to the development of atherosclerosis.
Hayrapetian et al. Dec, 2021 [16], LA, USA.	114 (0) 74±7 y	Pts with NaF-PET/CT bone scan for prostate cancer/ bone lesion and ^{82}Rb myocardial PET for CAD/ chest pain within same year.	NaF	Proximal coronary arteries.	Retrospective analysis to study the association between NaF uptake and severity of coronary stenosis.	TBRmax and CT visible lesions of > 130 Hounsfield units quantified by TeraRecon CT software.	Pts with ischemic stress ^{82}Rb PET had significantly higher coronary NaF uptake than pts with normal perfusion (P < .01). Among 41 pts, who underwent coronary angiography, per-vessel NaF uptake in both obstructive and non-obstructive coronary arteries was significantly higher than in normal coronary arteries (P < .05), regardless of the severity of coronary calcification. Poor correlation between calcification and NaF uptake in coronary arteries (r=0.41).	Authors end by stating that “coronary arterial NaF uptake is associated with coronary stenosis severity….” However, it’s atherosclerosis and not stenosis severity what this is about.

CAD = coronary artery disease; CV = cardiovascular; CVD = cardiovascular disease; DM = diabetes mellitus; FDG = 18F-fluorodeoxyglucose; NaF = 18F-sodium fluoride; pts = patients; PAD = peripheral arterial disease; PXE = pseudoxanthoma elasticum; RA = rheumatoid arthritis; SUV = standardized uptake value; TBR = target-to-background ratio; Y = years.

Journal of Imaging

Table S3. Apr 2020 – Mar 2022 Studies on NaF Uptake in Vulnerable, High-Risk and Ruptured Plaque.

First au-thor, time (Ref. #) Site	Patients, n (females) Age in years Mean±SD or range	Material	Tracer	Arterial seg-ment	Purpose	Quantification	Main Findings	Comments
Ashwa-thanarayana et al. Jul 2020 [17] Chandigarh, India.	MI group: 24 (4) 49.3±13.2 y and CSA group 17 (4) 50.8±13.2 y	MI group, all with acute ST-elevation in-farcts, PET/CT within 2 days of event and within 3 days of event In CSA group.	NaF	Coronary arter-ies	NaF PET/CT to character-ize plaques in ST-eleva-tion myocardial infarction (MI) and chronic stable angina (CSA) patients.	SUVmax and TBRmax of lesions	Culprit plaques in MI pts had significantly higher SUVmax (1.6; IQR 0.6 vs 1.3; IQR 0.3, P = 0.03) and TBR (1.4; IQR 0.6 vs 1.1; IQR 0.4, P = 0.006) than culprit plaques of CSA. Pre-interventional cul-prit plaques of MI group (n = 11) revealed higher SUVmax (P = 0.007) and TBR (P = 0.008) values than culprit CSA plaques. Cul-prit plaques showed significantly higher SUVmax (P = 0.006) and TBR (P = 0.0003) than non-culprit plaques in MI group, but no significant difference in CSA group. With median TBR cutoff value of 1.4 in MI culprit plaques, 6/7 plaques (85.7%) among the event prone non-culprit lesions had TBR values > 1.4 in CSA group.	
Majeed et al. Epub 2020 Dec 15 [18] Perth, Australia.	62 (9) 61±9 y	Prospectively re-cruited pts with acute coronary syndrome (ACS).	NaF	Proximal coro-nary arteries	Coronary NaF uptake and high-risk plaque features on intra-coronary optical coherence tomography (OCT) and CT-angi-ography	TBRmax = SU-Vmax/SUVmean blood pool.	Coronary segments with elevated NaF uptake had higher lipid arc (74º vs 48º), higher prevalence of macrophages (62% vs 39%), and lower plaque free wall (50º vs 94º) on OCT, and higher total plaque burden and dense calcified plaque burden on CT-angio, when compared to with NaF negative segments. Coronary NaF uptake contributes to further characterize coronary plaques and according to the authors to refine risk stratification of pts with ACS.	It remains to be documented by follow-up studies to what degree coronary NaF uptake "refine risk stratification of pts with ACS". However, ref 26 clearly points in this direction too.
Mechtouff et al. Nov 2020 [19] Lyon, Bron, St. Etienne, France.	6 (1) 72 (59-78) y sympto-matic and 6 (2) 27 (63-75) y asympto-matic.	Pts with symptomatic (transitory ischemic attack or minor stroke ≤ 15 days) or asymptomatic carotid stenosis ≥ 50%.	NaF	Carotids and aortic arch, ostium of bra-chiocephalic trunk, left sub-clavian artery, left common carotid artery.	NaF uptake vs morpho-logical criteria of vulnera-bility and in relation to TNAP which by hydro-lyzing inorganic pyro-phosphate contributes to formation of hydroxyap-atite.	TBRmax (SUVmax divided by blood pool activity in su-perior vena cava).	Culprit plaques = plaques responsible of symptoms ≤ 15 days, nonculprit = contralateral plaques of symptomatic pts or plaques of asymptomatic pts. Three ROIs centered on area of highest up-take in the plaque. If no plaque, NaF uptake on contralateral ca-rotid in proximal 1 cm of internal carotid artery distal to bifurca-tion was quantified. NaF uptake higher in culprit than nonculprit plaques (median TBR 2.6 [2.2-2.8] vs 1.7 [1.3-2.2]; P = 0.03) but not associated with morphological criteria of vulnerability on MRI. Positive correla-tion between NaF uptake and calcium plaque volume and ratio but not with circulating tissue-nonspecific alkaline phosphatase activity and inorganic pyrophosphate levels. NaF uptake in the other arterial walls did not differ between symptomatic and asymptomatic patients.	In 4 pts with stroke, intense NaF uptake was noted (TBRmedian 6.2 vs. 0.2 in con-tralateral non-infarcted brain).
Wurster et al. 2022 Jan 7 [20] Berlin, Göttingen, etc., Germany, London, UK, San-tiago, Chile.	21 (4) 67±7 y	Pts scheduled for in-vasive coronary an-gio. Multiple risk fac-tors (hypertension 86%, hyperlipidemia 81%, diabetes 43%).	NaF	8 coronary seg-ments: prox, mid, distal RCA, LM, prox, mid, distal	Feasibility and diagnostic potential of NaF and gadobutrol enhanced dual-probe PET/MR in CAD pts. Arbitrary	TBRmax and con-trast-to-noise ratio (CNR) values (MRI, gadobutrol) for each coronary seg-ment.	High-risk plaques, i.e. ,calcified and non-calcified thin-cap fi-broatheromas were predominantly located in segments with a TBRmax >1.28 (P = 0.012). Plaques containing a lipid core on OCT, were more frequently detected in segments with a TBRmax >1.25 (P < 0.001). TBRmax values significantly correlated with maximum calcification thickness (P = 0.009), while fibrous cap	No real clinically useful new knowledge.

First au-thor, time (Ref. #) Site	Patients, n (females) Age in years Mean±SD or range	Material	Tracer	Arterial seg-ment	Purpose	Quantification	Main Findings	Comments
				LAD; prox, dis-tal LCX.	PET/CT TBR thresholds for adverse coronary events.	OCTwas used as reference.	thickness was significantly less in segments with a TBRmax >1.28 (P = 0.044). Above a TBRmax threshold of >1.28, CNR values sig-nificantly correlated with the presence of calcified thin-cap fi-broatheromas (P = 0.032).	
Kaczynski et al. Oct 2022 [21] Edinburgh, UK, Toronto, Canada, LA, USA.	110 (40) 68±10 y	Pts > 40 y with neuro-vascular event < 14 days. 34 prior cere-brocascular disease, 26 amaurosis fugax, 54 TIA, 30 stroke.	NaF	Most severe culprit stenosis, contralateral carotid and aortic arch.	Association of NaF activ-ity and culprit carotid plaque in acute neurovas-cular syndrome.	Visual NaF in cul-prit and non-culprit carotid lesions. Quantification with TBRmean and TBRmax.	Culprit carotids had greater stenoses (≥50% stenosis: 30% vs 15%, P = .02; ≥70% stenosis: 25% vs 4.5% , P < .001) and increased prev-alence of MRI-derived adverse plaque features, including intra-plaque hemorrhage (42% vs 23%; P = .004), necrotic core (36% vs 18%; P = .004), thrombus (7.3% vs 0%; P = .01), ulceration (18% vs 3.6%; P = .001), and slightly higher NaF uptake (TBRmax 1.38 vs 1.26; P = .04). Higher NaF positively associated with necrosis, in-traplaque hemorrhage, ulceration, and calcification and inversely associated with fibrosis (P = .04 to P < .001). Carotid stenosis ≥70% and MRI-derived adverse plaque characteristics were both associated with the culprit versus nonculprit carotid vessel.	

ACS = acute coronary syndrome; CAD = coronary artery disease; CNR = contrast to noise ratio; CSA = chronic stable angina; CV = cardiovascular; CVD = cardiovascular disease; DM = diabetes mellitus; FDG = 18F-fluorodeoxyglucose; LAD = left anterior descending; LCX = left circumflex; LM = left main; MI = myocardial infarct; NaF = 18F-sodium fluoride; NASCET = North American Symptomatic Carotid Endarterectomy Trial; OCT = optical coherence tomography; pts = patients; RCA = right coronary artery; SUV = standardized uptake value; TBR = target-to-background ratio; TIA = transient ischemic attack; TNAP = tissue non-specific alkaline phosphatase ; Y = years.

Journal of Imaging

Table S4. Apr 2020 – Mar 2022 Studies on Association Between Arterial NaF Uptake and Risk Factors.

First author, time (Ref. #) Site	Patients, n (females) Age in years Mean±SD or range	Material	Tracer	Arterial segment	Purpose	Quantification	Main findings	Comments
Rojulpote et al. Jun 2020 [22] Philadelphia, US & Odense, Denmark.	20 (8) 41.6±13.8 y	Asymptomatic healthy individuals with no smoking, diabetes or dyslipidemia and with normal blood pressure.	NaF	Global cardiac uptake by manual segmentation.	Is coronary artery microcalcification in healthy controls without macroscopic CT calcification related to blood pressure?	Cardiac average SUVmean (aSUVmean)	After adjusting for age and gender, diastolic and mean arterial blood pressures were independent 'predictors' of higher global cardiac NaF uptake.	
Patil et al. Aug 2020 [23] Philadelphia, US & Odense, Denmark.	68 (33) 41.7±13. 5 y	Healthy, non-diabetic individuals.	NaF	Entire heart.	Triglycerides (TG) /high density lipoprotein (HDL) ratio as a marker of insulin resistance and atherosclerosis: Does this ratio correlate positively with global cardiac SUVmean?	Global cardiac aSUVmean	Univariate analysis: positive linear association of TG/HDL ratio and global cardiac aSUVmean (r=0.244, B=0.047, P=0.045). Multivariate analysis adjusted for age, gender, systolic blood pressure, diastolic blood pressure, smoking status, total cholesterol, low-density lipoprotein, and fasting plasma glucose: TG/HDL ratio was independently associated with global cardiac aSUVmean (B=0.060, 95% CI: 0.007-0.114, P=0.027). Conclusion: There was a positive correlation between TG/HDL ratio with global cardiac microcalcification assessed by NaF-PET/CT imaging.	First study examining the association of TG/HLD ratio and the burden of coronary atherosclerosis in asymptomatic non-diabetic individuals.
Gonuguntla et al. Dec 2020[24] Philadelphia, US & Odense, Denmark.	40 (22) 55±11.9 y	High risk pts.	NaF	Entire heart.	Global cardiac NaF uptake compared with CHADS2 and CHA2DS2-VASc scores used to estimate risk of stroke in pts with atrial fibrillation.	Global cardiac aSUVmean	The sample consisted of subjects with a mean aSUVmax of 2.9 ± 1.4, aSUVmean was 0.8 ± 0.2, CHADS2 0.9 ± 0.6 (Range: 0-3), CHA2DS2-VASc 1.8 ± 1.3 (Range: 0-5). Direct correlation between global cardiac aSUVmean and CHADS2 score (r=0.58, P≤0.0001) and also between global cardiac aSUVmean and CHA2DS2-VASc (r=0.37, P=0.01). Patients with higher CHADS2 and CHA2DS2-VASc scores had a higher atherosclerotic burden and could be at greater risk of cardiovascular events.	
Borja et al. Dec 2020 [25] Philadelphia, US & Odense, Denmark	61 (32) 53.4±8.9 y	Pts >40 y with unknown risk split in 4 ASCVD 10-year risk groups: low (<5%, n=32), borderline (5-7.4%, n=10), intermediate (7.5-19.9%, n=17), and high risk (≥20%, n=2).	NaF	Entire heart.	Is the ACC/AHA ASCVD risk score used clinically ahead of older risk scores to examine topics from drug mechanisms correlated to global cardiac microcalcification?	Global cardiac aSUVmean	Global cardiac aSUVmean stratified by groups estimated by 10-year ASCVD risk score were 0.67±0.09 for low risk (n=32), 0.70±0.11 for borderline risk (n=10), 0.72±0.10 for intermediate risk (n=17), and 0.78±0.10 for high risk (n=2). ASCVD risk score was significantly correlated to aSUVmean (r=0.27, P=0.03).	Note: pts below 40 y of age are not accounted for by the ASCVD score.<br><br>First (?) study to compare ASCVD risk scores to cardiac plaque burden as assessed by NaF-PET/CT.
Paydary et al. Feb 2021 [26], Odense, Denmark & Philadelphia, USA.	80 (?) healthy controls aged 44.5 (22-75) y and 44 (?) chest pts aged 59.5 (23-75) y	Reanalysis of prospectively collected material.	NaF	Ascending, arch, descending parrs of thoracic aorta and their total (TA).	Association with age and ACS as potential "predictor" of unfavorable CVD risk compared with other values.	aSUVmax, aSUVmean and Alavi-Carlsen Score (ACS), see reference (59).	aSUVmax, aSUVmean, and ACS were significantly higher in pts than controls, all correlated with age. Correlation of aSUVmean with age was significant in both controls (r = 0.32, p = 0.04) and pts (r = 0.64, p < 0.001). ACS of TA was a stronger predictor of Framingham Risk Score than aSUVmax and aSUVmean.ACS was a significant predictor of unfavorable CVD risk profile as compared with other values (odds ratio = 1.006, 95% CI = 1.000–1.013, p = 0.05).	

First author, time (Ref. #) Site	Patients, n (females) Age in years Mean±SD or range	Material	Tracer	Arterial segment	Purpose	Quantification	Main findings	Comments
Borges-Rosa et al. 2021 Apr 6. [27] Coimbra, Portugal.	34 (13) 63.5±7.8 y	High CV risk individuals with previous CV events.	NaF	Entire heart.	To evaluate global cardiac microcalcification activity with NaF, as a measure of unstable microcalcification burden, in high CV risk pts.	Cardiac global molecular calcification score (GMCS) = SUVmean. Note: valves included.	Median GMCS was 320.9 (240.8-402.8). Individuals with more than five CV risk factors (50%) had increased GMCS [356.7 (321.0-409.6) vs. 261.1 (225.6-342.1), P = 0.01], which was positively correlated with predicted fatal CV risk by SCORE (rs = 0.32, P = 0.04). Positive correlation between GMCS and weight (rs = 0.61), BMI (rs = 0.66), abdominal perimeter (rs = 0.74), thoracic fat volume (rs = 0.47), and epicardial adipose tissue (rs = 0.41), all with P ≤ 0.01. No correlation between GMCS and coronary calcium score or between coronary artery wall NaF uptake and coronary calcium score. Conclusions: In a high CV risk group, the global cardiac microcalcification burden is related to CV risk factors, metabolic syndrome variables and cardiac fat. Cardiac GMCS is a promising risk stratification tool, combining a straightforward and objective methodology with a comprehensive analysis of both coronary and valvular microcalcification.	
Castro et al. Oct 2021 [28] Philadelphia, US & Odense, Denmark	128 (63) 48±14 y	Pts with mixed cardiovascular risk.	NaF	Left common carotid artery.	Association between left common carotid NaF uptake and CV/ thromboembolic risk.	Left carotid NaF as SUVmax on each axial slice. aSUVmax over all slices.	aSUVmax over all slices correlated with 10-year risk of cardiovascular events estimated by the Framingham model, CHA2DS2-VASc score, and level of physical activity (LPA); aSUVmax significantly higher in pts with increased risk of cardiovascular and thromboembolic events, and significantly lower in pts with greater LPA. Age, BMI, hypertension and LPA were independent associations of aSUVmax.	

ASCVD = Atherosclerotic Cardiovascular Disease; aSuv = average SUV; BMI = body mass index; CAD = coronary artery disease; CV = cardiovascular; CVD = cardiovascular disease; DM = diabetes mellitus; FDG = 18F-fluorodeoxyglucose; LPA = level of physical activity; NaF = 18F-sodium fluoride; pts = patients; SUV = standardized uptake value; TA = total (thoracic) aorta; TBR = target-to-background ratio; Y = years.

Table S5. Apr 2020 – Mar 2022 Studies on NaF Uptake and Disease Progression or 'Prediction' of Events.

First author, time (Ref. #) Site	Patients, n (females) Age in years Mean±SD or range	Material	Tracer	Arterial segment	Purpose	Quantification	Circumstances/finding	Comments
Kwiecinski et al. 2020 Jun 23[29] Cedars-Sinai, LA, USA & Edinburgh, UK.	293 (47) 65±9 y	Pts with known CAD.	NaF	Proximal coronaries.	Coronary NaF uptake for prediction of myocardial infarction – compared to current risk stratification.	TBRmax = SUVmax in proximal coronary arteries divided by blood pool activity in right atrium.	After 42 (31-49) months of follow-up, fatal and non-fatal myocardial infarction had occurred only in pts with abnormal NaF uptake (20/203 vs 0/90 in pts without abnormal coronary NaF uptake). Pts with increased NaF uptake had >7-fold increase in myocardial infarction incidence independent of age, gender, CV risk factors, segment involvement scores, presence of coronary stents, number of vessels with significant stenosis, coronary calcium scoring, REACH and SMART scores, Duke index, and recent MI.	Suggests strongly that abnormal coronary NaF uptake carries a significant risk of future myocardial infarction!
Bellinge et al. Jan 2021 [30] Perth, Western Australia.	41 (15) 65±7.1 and CCS≥10 & 10 (6) 61.2±8.4 and CCS=0	Diabetic pts with no history of CAD. All had NaF-PET/CT and CT CCS scans at baseline; 41 had repeat CT CCS scans after 2.8±0.5 y. 26/41 of high-risk and 4/10 of low-risk cohort received statins at baseline.	NaF	163 coronary arteries analyzed (one obscured by pacemaker lead).	Can localized coronary artery NaF uptake predict development of new CT detectable calcifications at least 2 years later?	CCS in Agatson units; NaF uptake in 2D ROIs around coronary arteries on3 mm consecutive axial slices to get a SUVmax, which adjusted for right atrial blood activity yielded TBRmax.	The proportion of "CCS progressors" was higher among NaF positive than NaF negative arteries at baseline (86.5% vs 52.3%, p<0.001. NaF positive disease was an independent "predictor" of subsequent CCS progression (odds ratio 2.92 [95% CI 1.32-6.45], p=0.008. All subjects (15/15) with ≥ 2 NaF positive coronary arteries progressed in CCS. Note: All pts had had previously undergone CT CCS screening and NaF PET/CT as baseline assessment for the effect of Vitamin-K1 and Colchicine on Vascular Calcification Activity in subjects with Diabetes Mellitus (ViKCoVaC) trial. Consecutive pts invited to the present project 2 y after completion of VIKCOVAC trial, see (39).	Baseline NaF uptake did not 'predict' development of new CT detectable coronary artery calcification as stated in the title. Shown was only a statistically significant association between NaF positivity at baseline and CCS development.
Doris et al. 2020 Dec [31] Edinburg, UK, Cedars-Sinai, LA, USA, Christchurch, NZ.	183 (37) 66 (59-71) y	Pts with multivessel disease with NaF PET/CT and CT angio at baseline and repeat CT angio at one year.	NaF	Proximal coronary arteries.	To investigate the relationship between NaF uptake and progression of coronary calcification in patients with clinically stable coronary artery disease.	TBRmax.	In a total of 183 participants (median age 66 years, 80% male), 116 (63%) patients had increased NaF uptake in at least one vessel. Individuals with increased NaF uptake demonstrated more rapid progression of calcification compared with those without uptake (change in calcium score, 97 [39–166] versus 35 [7–93] AU; P<0.0001). The calcium score only increased in coronary segments with NaF uptake (from 95 [30–209] to 148 [61–289] AU; P<0.001) and remained unchanged in segments without NaF uptake (from 46 [16–113] to 49 [20–115] AU; P=0.329). Baseline coronary NaF TBRmax correlated with 1-year change in calcium score, calcium volume, and calcium mass (Spearman ρ=0.37, 0.38, and 0.46, respectively; P<0.0001 for all). At the segmental level, baseline NaF activity was an independent predictor of calcium score at 12 months (P<0.001).	

First author, time (Ref. #) Site	Patients, n (females) Age in years Mean±SD or range	Material	Tracer	Arterial segment	Purpose	Quantification	Circumstances/finding	Comments
							However, at the patient level, it was not independent of age, sex, and baseline calcium score (P=0.50).	
Hu et al. Feb 2021 [32] Shanghai, China.	ApoE-/- mice and control mice.	Mice examined at 12, 20, and 30 weeks of age, n=3 at each time point in each group.	NaF	Excised aorta.	To dynamically monitor the progression of atherosclerotic plaques in ApoE-/- mice with NaF PET imaging.	Semi-quantitative analysis of NaF-avidity of atherosclerotic plaques on μPET.	Aortic uptake of NaF was gradually increased with each weekly extension. Compared with the ApoE-/- mice at the age of 12 weeks and 20 weeks, the levels of lipoprotein, inflammatory cytokines, and calcification factors were higher at 30 weeks. In Oil Red O, HE, and alizarin red (calcium) staining, the extent of the lipid area and calcification increased with time. Correlation analysis showed that the uptake of NaF in the aorta was related to the extent of calcification.	
Fiz et al. Feb 2021 [33] Milan & Genoa, Italy.	71 (27) 71±6.9 y	Pts who underwent 2 NaF PET/CT scans due to proven or suspected bone metastases; statin medication not specified.	NaF	Infrarenal aorta.	Does NaF uptake (1) identify early uncalcified plaque and (2) does NaF uptake at plaque or vessel level correlate with subsequent increase in plaque calcium content?	Apparantly averageTBRmean for the entire aortic ROI, calculated as average SUVmean divided by inferior vena cava activity.	2consecutive NaF-PET/CTs (PET1/PET2) performed 15.5 ± 9.7 months apart. In PET1, non-calcified NaF hot spots were identified. Their mean/max HU was compared with those of a non-calcified control region (CR) and with corresponding areas in PET2. TBRmean density (HU) and calcium score (CS) of calcified atherosclerotic plaques in PET1 were compared with those in PET2. Results: Hot spots in PET1 (N = 179) had a greater HU than CR (48 ± 8 vs 37 ± 9, P < .01). Mean hot spots HU increased to 59 ± 12 in PET2 (P < .001). New calcifications appeared at the hot spots site in 73 cases (41%). Baseline atherosclerotic plaque's (N = 375) TBR was proportional to percent HU and CS increase (P < .01 for both). Aortic CS increased (P < .001); the whole-aorta TBR in PET1 correlated with the CS increase between the baseline and the second PET/CT (R = .63, P < .01). Conclusions: NaF-PET/CT depicts early stages of plaque development and tracks evolution over time.	Pts imaged on two different scanners, i.e., Siemens and GE; however not stated if pts were imaged on the same scanner on both occasions.
Brodsky et al. Jan-Apr 2021 [34] Philadelhia, USA & Odense, Denmark.	Look right	Healthy females (n=8, 52±10 y, BMI 24±1.7kg/m2) and healthy males (n=15, 50±10 y, BMI 27±2.9kg/m2)	NaF	Entire heart semi-automated manual segmentation.	Are 2-year changes in coronary microcalcification by NaF PET/CT associated with baseline subject characteristics?	SUVmean and SUVmax and mean HU in same ROI.	Percent change in SUV between the two time points were correlated against baseline age, BMI, cardiovascular risk factors, and blood chemistry. In males, percent change in SUVmean over the two year period was positively correlated with baseline BMI (r=0.85, P<0.0001) and systolic blood pressure (r=0.65, P=0.0082). These baseline values were not significantly correlated with SUVmax in either gender. Lack of such associations in females could be due to low sample size (n=8).	

First author, time (Ref. #) Site	Patients, n (females) Age in years Mean±SD or range	Material	Tracer	Arterial segment	Purpose	Quantification	Circumstances/finding	Comments
Lillo et al. Jun 2021 [35] Málaga, Spain.	14 (?) Age not stated.	PXE pts with 2 NaF PET/CT scans 2 years apart.	NaF	Carotids, 4 aortic sections, iliac, femoral, popliteal arteries.	Changes over time (2 years) in skin and arterial NaF depositions.	SUVmax and SUVmean and TBRmax and TBRmean. CT calcium deposits by score, volume, mass.	CT-detect able calcium deposits as a mean of all vascular territories increased in the group as a whole, albeit due to increases in two pts only, i.e., unchanged score in 12. SUVmax and SUVmean remained unchanged in all territories in the group as a whole, whereas TBRmax and TBRmean decreased by 24% and 19%, respectively (p=0.04 for both). NaF uptake in skin deposits did not change.	Strange that SUVmean didn't decrease while TBRmean did. Description of segmentation is lacking making further interpretation impossible.
Reijrink et al. Sep 2021 [36] Groningen, The Netherlands.	10 (3) 63 (59-69) y	Type 2 diabetic patients without glucose lowering drugs and a severe CV history.	FDG & NaF	10 arteries in 4 segments: (1) Carotids, 2) asc. + arch, (3) desc. + abd. aorta, (4) iliac + femoral arteries.	Prospective correlation between tracers over time and whether they are prospectively (FDG) and retrospectively (NaF) related to progression.	TBRmax (called $_{mean}$TBR by authors) using SUVmax of ten arteries divided by SUVmean of caval vein. CT calcification as calcified plaque (CP) score. Carotid-femoral PWV.	Baseline $_{mean}$TBR FDG was strongly correlated with five-year follow-up $_{mean}$TBR NaF (r = 0.709, P = .022). $_{mean}$TBR NaF correlated positively with ΔCPscore, CPscore at baseline, and follow-up (r = 0.845, P = .002 and r = 0.855, P = .002, respectively), but not with %change in CPscore and PWV.	Small study suggesting initial FDG uptake is associated with arterial NaF uptake 5 years later and that NaF uptake (measured after 5 years) is associated with calcium score and PWV at baseline and 5 years, but not with 5-year change of these.
Piri et al. Oct 2021 [37] Odense, Denmark.	29 (13) 51 (21–75) y healthy controls and 20 (10) 57 (23–67) y angina pectoris pts.	Healthy controls and angina pectoris scanned 2 years apart.	NaF	Carotids and arch, thoracic and abdominal aorta.	2-year changes in NaF uptake and CT calcification.	SUVmean and SUVtotal ± partial volume correction (pvc).	Insignificant tendencies were higher NaF uptake in angina patients at both time points with less uptake in healthy subjects and higher uptake in angina patients after 2 years. Thus, aortic pvcSUVmean of angina patients was 1.14 ± 0.35 and 1.29 ± 0.71 at baseline and after 2 years vs. 0.99 ± 0.31 and 0.95 ± 0.28 in healthy subjects. Similar pattern for carotid pvcSUVmean. Baseline NaF uptake could not predict a change in CT-calcification after 2 years. 2-year changes in both groups very small suggesting that atherosclerotic process is slow.	
Kitagawa et al. Apr 2022 [38] Hiroshima, Japan	15 (1) 64±8 y	Pts with ≥ 1 coronary artery atherosclerotic lesion. Total 55 lesions of which 51 analyzed at baseline and follow up after 45±8 months.	NaF	ROIs defined as visually detected coronary CT lesions.	Change in NaF uptake in coronary CT calcifications and potentially impacting factors.	TBRmax with two cut offs: 1.0 and 1.28. R-TBRmax = ratio of follow-up to baseline; value > indicating progression.	Mean R-TBRmax 0.96±21 indicating no change in NaF uptake in 45 months (range 37-59). CT-based lesion features (location, obstrruction, plaque type, high-risk features) did not correlate with R-TBRmax. Baseline TBRmax correlated with higher follow-up TBRmax, and presence of DM (6/15 pts) correlated with higher follow-up TBRmax and (slightly) elevated R-TBRmax. 63% of NaF positive lesions at baseline were positive at follow-up.	Small material, next generation PET/CT scanner used for follow-up scan, NaF uptake independent of CT-detectable lesions not studied.
Kwiecinski et al. May 2022 [39], CA, USA, Edinburgh, UK	48 (4) 68±8 y with prior CABG,	Pts with established CAD with and without prior CABG	NaF	NaF in 'entire' coronary bed;	NaF PET to detect grafts vasculopathy and investigate influ-	Coronary microcalcification activity (CMA) = sum of NaF activity,	Among 154 grafts, 37 (24%) showed vasculopathy on CT and 20 (13%) were occluded. All arterial and the majority of 120/128 (94%) of venous grafts showed no NaF uptake, which was seen in only 8 saphenous grafts (in 7 patients).	Rare NaF uptake in bypass grafts. Bypassed patients have more aggressive CT-calcification, apparently detached

First author, time (Ref. #) Site	Patients, n (females) Age in years Mean±SD or range	Material	Tracer	Arterial segment	Purpose	Quantification	Circumstances/finding	Comments
	mean 2.7 y previously vs. 48 (4) 69±6 y without prior CABG			autoradiographs of saphenous vein grafts.	ence of CABG on native CAD and progression.	where SUV > 'corrected' RA blood activity.	Bypassed native coronary arteries had 3 times higher CMA than non-bypassed arteries and greater progression of 1-year calcium scores, an effect largely confined to native coronary plaques proximal to the graft anastomosis. Thus, microcalcification is not a common feature of graft vasculopathy, but bypassed patients have more aggressive disease.	from modest NaF uptake – late in the course when statins have had time to work?
Fletcher et al. Jul 2022 [40] Edinburgh, UK, Naples, Italy, Warsaw, Poland, etc.	461 (98) 69.98±8.48 y	Pts with established CVD.	NaF	Large part of coronary arteries and ascending aorta and arch.	Can thoracic aortic NaF uptake improve identification of pts at the highest risk of ischemic stroke?	Global SUVmean of coronary arteries and the ascending and arch part of the thoracic aorta compared to CT calcium scores.	Large retrospective analysis of 4 materials. After 12.7 months, progression of thoracic aortic calcium volume correlated with baseline thoracic aortic NaF activity (n = 140; r = 0.31; P = 0.00016). In the 461 patients, 23 (5%) patients experienced an ischemic stroke and 32 (7%) a myocardial infarction after 6.1 of follow-up. High thoracic aortic NaF activity was strongly associated with ischemic stroke (HR: 10.3; P = 0.00017), but not myocardial infarction (P = 0.40). Conversely, high coronary 18F-sodium fluoride activity was associated with myocardial infarction (HR: 4.8; P = 0.00095) but not ischemic stroke (P = 0.39). Thoracic NaF activity was the only variable associated with ischemic stroke (HR: 8.19 [95% CI: 2.33-28.7], P = 0.0010). Conclusions: In patients with established cardiovascular disease, thoracic aortic 18F-sodium fluoride activity is associated with the progression of atherosclerosis and future ischemic stroke.	
Dai et al. Oct 2022 [41] Philadelphia, USA.	36 Age not reported.	Men with NaF PET/CT scans fore evaluation of previous prostate cancer and liquid panel results.	NaF	Manual sections of aortic arch and thoracic aorta.	To find noninvasive, cost-efficient, readily available metrics for predicting vascular calcification severity.	Average SUVmean of all axial slices of the thoracic aorta.	Retrospective study. Correlation analyses between SUVs and calculated atherogenic indices: Castelli's Risk Index I (r = 0.63, p < 0.0001), Castelli's Risk Index II (r = 0.64, p < 0.0001), Atherogenic Coefficient (r = 0.63, p < 0.0001), Atherogenic Index of Plasma (r = 0.51, p = 0.00152), and standalone high-density lipoprotein (HDL) cholesterol (r = -0.53, p = 0.000786), all associated with aortic NaF. However, no possibility of characterizing individual patients with high likelihood.	Three weak correlations with large scatter, significant due to one outlier. One fair correlation. No clinically useful prediction.

CABG = Coronary artery bypass graft; CAD = coronary artery disease; CCS = Coronary calcium score; CP = calcified plaque; CR = control region; CS = calcium score; CV = cardiovascular; CVD = cardiovascular disease; DM = diabetes mellitus; FDG = 18F-fluorodeoxyglucose; HU = Hounsfield unit; MI = myocardial infarction; NaF = 18F-sodium fluoride; pts = patients; pvc = partial volume corrected; PWV = pulse wave velocity; PXA = pseudoxanthoma elasticum; RA = right atrium; ROI = region of interest; SUV = standardized uptake value; TBR = target-to-background ratio; Y = years.

Table S6. Apr 2020 – Mar 2022 Studies on Anti-Atherosclerotic Intervention Evaluated by NaF PET/CT.

**First author, time (Ref. #) Site**	**Patients, n (females) Age in years Mean±SD or range**	**Material**	**Tracer**	**Arterial segment**	**Purpose**	**Quantification**	**Circumstances/finding**	**Comments**
Hsu at al. Oct 2020 [42] LA, USA.	9 + 9 mice	2 groups of hyperlipidemic ($Apoe^{-/-}$) mice, one moved ad-lib; the other had progressive treadmill regimen for 9 weeks.	NaF	Aortic root.	Can exercise transform vascular calcium deposits to a more stable morphology?	(1) NaF density = NaF uptake as % injected dose normalized to deposit volume ($\%IDcc^{-1}$) and (2) total surface area, = total NaF as % injected dose (%ID).	In vivo NaF μPET/μCT imaging at start and end of exercise regimen demonstrated that while aortic calcification progressed similarly in both groups based on μCT, the fold change in NaF density was significantly less in the exercise group. Histomorphometric analysis of aortic root calcium deposits showed that the exercised mice had a lower mineral surface area index than the control group. The exercise regimen also raised serum PTH levels twofold. Thus, these findings suggest that weeks-long progressive exercise alters the microarchitecture of atherosclerotic calcium deposits by reducing mineral surface growth, potentially favoring plaque stability.	Exercise like statin therapy appears to reducing mineral surface area so that observed decrease or slowed increase in NaF uptake may be due to structural Ca-deposit changes rather than removal of microcalcification.
Florea et al. 2021 Jan 30 [43] Achen and Göttingen, German and Maastricht, NL	5 wild type mice and 20 ApoE-/- mice split in 4 groups of each 5 mice	Wild type on chow diet 12+12 weeks; ApoE-/- groups: All on Western type diet for 12 weeks followed by a) chow diet, b) Western type diet, c) MK-7 diet or d) Warfarin diet for another 12 weeks.	NaF	Left ventricle, aortic root, aortic arch	To assess the ability of Na[18F]F to monitor therapy and disease progression in a unitary atherosclerotic mouse model.	TBRmax	The Warfarin group presented spotty calcifications on the CT in the proximal aorta. All of the spots corresponded to dense mineralization on the von Kossa staining. After the control, the MK-7 group had the lowest NaF uptake. The advanced and Warfarin groups presented the highest uptake in the aortic arch and left ventricle. The advanced stage group did not develop spotty calcifications, however, NaF uptake was still observed, suggesting the presence of micro-calcifications. In a newly applied mouse model, developing spotty calcifications on CT exclusively in the proximal aorta, NaF seems to efficiently monitor plaque progression and the beneficial effects of vitamin K on cardiovascular disease.	
Dietz et al Jul 2021 [44] Monaco.	1 (1) 64 y	Asymptomatic man with non-severe hypercholesterolemia.	NaF	Proximal coronary arteries		Coronary Agatston score and SUVmax by NaF PET/CT.	After 6-months on 10 mg rosuvastatine and 75 mg asprin per day and advice on diet and exercise, CT showed unchaged Agatston score of coronary plaques, while NaF part of PET/CT demonstrated halving of SUVmax values without no new hot spots.	Decrease in NaF uptake also visually apparent.
Zhang et al. Oct 2021 [45] Beijing, China.	10+10 rabbits in 2 groups: Atherosclerosis and Atorvastatin	Atorvastatin 5 mg/kg/d. All rabbits had abdominal aortic dilated balloon operation after 2 weeks of feeding and then	NaF	Abdominal aorta	Examine effect of atorvastatin on plaque calcification by matching results obtained by NaF	SUVmean and SUVmax. Plaque area, macrophage number and calcification were measured. Data	SUVmean (0.725 ± 0.126 vs. 0.603 ± 0.071, P , 0.001) and SUVmax (1.024 ± 0.116 vs. 0.854 ± 0.091, P = 0.001) significantly increased in the atherosclerosis group, but only slightly increased in the atorvastatin group (0.616 ± 0.103 vs. 0.613 ± 0.094, P =	The authors concluded that anti-inflammatory activity of atorvastatin may promote macrocalcification but not

Journal of Imaging

First author, time (Ref. #) Site	Patients, n (females) Age in years Mean±SD or range	Material	Tracer	Arterial segment	Purpose	Quantification	Circumstances/finding	Comments
	group on cholesterol-enriched (2%) diet.	continuously fed on cholesterol-enriched diet for 16 weeks.			PET/CT with histologic data.	from pathological sections were matched with NaF uptake.	0.384; 0.853 ± 0.099 vs.0.837 ± 0.089, P = 0.001, respectively). Total calcium density significantly increased in atorvastatin treated vs. non-atorvastin treated rabbits (1.64 6 ±.90 vs. 0.49 ± 0.35, P = 0.001), but the microcalcification level was significantly lower. There were more microcalcification deposits in the areas with increased radioactive uptake of NaF.	microcalcification within atherosclerotic plaque.
Bellinge et al. 2021 Apr 6 [46] Perth, Western Australia.	154 (53) ~65±7 y	Pts with type 2 diabetes and CT coronary calcifications in a double-blind, placebo-controlled 2x2 factorial trial of 3 months duration.	NaF	Proximal coronary arteries and asc, arch, descending thoracic aorta.	Does vitamin-K1 or colchicine affect arterial calcification activity assessed by NaF PET?	TBRmax	The effect of Vitamin-K1 and Colchicine on Vascular Calcification Activity in subjects with Diabetes Mellitus (ViKCoVaC) trial with four treatments groups (placebo/placebo, vitamin-K1 [10 mg/day]/placebo, colchicine [0.5 mg/day]/placebo, vitamin-K1 [10 mg/day]/ colchicine [0.5 mg/day]). However, neither vitamin-K1 nor colchicine had a statistically significant effect on coronary TBRmax compared with placebo. There were no serious adverse effects reported with colchicine or vitamin-K1.	See also (27) above.
Bellinge et al. 2022 Jan 11 [47] Perth, Western Australia.	Same as [39]	Same material as [39]. 149 of the 154 completed baseline and follow-up studies.	NaF	Prox. coronary arteries and thoracic aorta.	Posthoc analysis of material in [39] with the TBRmax limit in [27] for each coronary and aortic segment.	Modified TBRmax with upper limit of normal = mean TBRmax + 2 SDs in 10 of non-treated DM cohort with 0 coronary calcium.	Posthoc analysis of ViKCoVaC trial data.Now, vitamin K1 supplementation independently decreased the odds of developing new NaF PET positive lesions in the coronary arteries (OR: 0.35; 95% CI: 0.16, 0.78; P = 0.010), aorta (OR: 0.27; 95% CI: 0.08, 0.94; P = 0.040), and in both aortic and coronary arteries (OR: 0.28; 95% CI: 0.13, 0.63; P = 0.002).	See also (27) above.
Jensen et al. Jul 2022 [48] Not caught in search	See next cell.	Atherosclerotic New Zealand White rabbits randomized to intervention- (n = 12) or placebo group (n = 11).	^{64}Cu-DOTATATE FDG NaF	Abdominal aorta	Effects of semaglutide on aorta of non-DM atherosclerosis rat model.	SUVmax and TBRmax. Autoradiography and histology.	PET/CT before and after 16-weeks of intervention with DOTATATE, FDG and Naf for imaging of activated macrophages, cellular metabolism and micro-calcifications, respectively. Significant reductions in uptake of DOTATATE and FDG in the semaglutide vs. placebo group, but similar NaF uptake in the two groups. Conclusion: Semaglutide reduces atherosclerotic inflammation by means of decreased activated macrophage activity.	
Bessueille et al. Jan 2023 [49]	See next cell.	4 cohorts of ApoE-deficient mice, from 10 weeks on high fat diet. First 2 cohorts used to characterize the abdominal	NaF	Aorta	Effect of TNAP inhibitor SBI-425 in ApoE-deficient mice, biocol-	NaF-μPET measuring SUVR using whole body SUV as reference.	Plaque calcification imaged in vivo with NaF-PET/CT, ex vivo with osteosense, and in vitro with alizarin red. TNAP activation preceded and predicted calcification in human and mouse	Complicated, well conducted extensive study applying multiple in vivo and ex vivo methodologies.

First author, time (Ref. #) Site	Patients, n (females) Age in years Mean±SD or range	Material	Tracer	Arterial segment	Purpose	Quantification	Circumstances/finding	Comments
Lyon/Bron France; Achen, Germany; CA, USA; Beirut, Lebanon.		aorta before using 2 other cohorts to study effects of SBI-425 vs no SBI-425.			lection of human carotid plaques and cultured human VSMCs and in skeleton and liver.	µCT for bone structure and NaF ROI.	plaques. TNAP inhibition prevented calcification in human VSMCs and in the ApoE-deficient mice. TNAP inhibition reduced blood levels of cholesterol and triglycerides, and protected from atherosclerosis, without impacting the skeletal architecture. Metabolomics analysis of liver extracts identified phosphocholine as a substrate of liver TNAP, who's decreased dephosphorylation upon TNAP inhibition likely reduced the release of cholesterol and triglycerides into the blood: Systemic inhibition of TNAP protects from atherosclerosis by ameliorating dyslipidemia, and preventing plaque calcification.	

CAD = coronary artery disease; CCS = Coronary calcium score; CV = cardiovascular; CVD = cardiovascular disease; DM = diabetes mellitus; FDG = 18F-fluorodeoxyglucose; GLP-1 = glucagon-like-peptide-1; NaF = 18F-sodium fluoride; PCR = polymerase chain reaction; pts = patients; SUV = standardized uptake value; TBR = target-to-background ratio; TNAP = tissue nonspecific alkaline phosphatase; VSMC = vascular smooth muscle cell; Y = years.

References

1. Derlin, T.; Richter, U.; Bannas, P.; Begemann, P.; Buchert, R.; Mester, J.; Klutmann, S. Feasibility of 18F-sodium fluoride PET/CT for imaging of atherosclerotic plaque. *J. Nucl. Med.* **2010**, *51*, 862–865. doi: 10.2967/jnumed.110.076471.
2. Høilund-Carlsen, P.F.; Piri, R.; Constantinescu, C.; Iversen, K.K.; Werner, T.J.; Sturek, M.; Alavi, A.; Gerke, O. Atherosclerosis Imaging with 18F-Sodium Fluoride PET. *Diagnostics (Basel)* **2020**, *10*, 852. doi: 10.3390/diagnostics10100852.
3. Høilund-Carlsen, P.F.; Sturek, M.; Alavi, A.; Gerke, O. Atherosclerosis imaging with 18F-sodium fluoride PET: state-of-the-art review. *Eur. J. Nucl. Med. Mol. Imaging* **2020**, *47*, 1538–1551. doi: 10.1007/s00259-019-04603-1.
4. Shamseer, L.; Moher, D.; Clarke, M.; Ghersi, D.; Liberati, A.; Petticrew, M.; Shekelle, P.; Stewart, L.A.; PRISMA-P Group. Preferred reporting items for systematic review and meta-analysis protocols (PRISMA-P) 2015: elaboration and explanation. *B.M.J.* **2015**, *350*:g7647. doi: 10.1136/bmj.g7647.
5. Zhuang, X.; Feng, Y.; Li, J.; Zhao, F.; Zhang, Y.; Chen, Y. A longitudinal 18F-fluorodeoxyglucose (18F-FDG) and 18F-sodium fluoride (18F-NaF) positron emission tomography/computed tomography (PET/CT) study in apolipoprotein E (ApoE) knockout rats fed with a Western diet. *Cardiovasc. Diagn. Ther.* **2021**, *11*, 39–49. doi: 10.21037/cdt-20-609.
6. Nogales, P.; Velasco, C.; Mota-Cobián, A.; González-Cintado, L.; Mota, R.A.; España, S.; Mateo, J.; Bentzon, J.F. Analysis of 18F-Sodium Fluoride Positron Emission Tomography Signal Sources in Atherosclerotic Minipigs Shows Specific Binding of 18F-Sodium Fluoride to Plaque Calcifications. *Arterioscler. Thromb. Vasc. Biol.* **2021**, *41*, e480–e490. doi: 10.1161/ATVBAHA.121.316075.
7. Omarjee, L.; Mention, P.J.; Janin, A.; Kauffenstein, G.; Pabic, E.L.; Meilhac, O.; Blanchard, S.; Navasiolava, N.; Leftheriotis, G.; Couturier, O.; Jeannin, P.; Lacoeuille, F.; Martin, L. Assessment of Inflammation and Calcification in Pseudoxanthoma Elasticum Arteries and Skin with 18F-FluroDeoxyGlucose and 18F-Sodium Fluoride Positron Emission Tomography/Computed Tomography Imaging: The GOCAPXE Trial. *J. Clin. Med.* **2020**, *9*, 3448. doi: 10.3390/jcm9113448.

8. Aaltonen, L.; Koivuviita, N.; Seppänen, M.; Kröger, H.; Tong, X.; Löyttyniemi, E.; Metsärinne, K. Association between bone mineral metabolism and vascular calcification in end-stage renal disease. *B.M.C. Nephrol.* **2022**, *23*, 12. doi: 10.1186/s12882-021-02652-z.
9. Wen, W.; Gao, M.; Yun, M.; Meng, J.; Zhu, Z.; Yu, W.; Hacker, M.; Yu, Y.; Zhang, X.; Li, X. Associations between coronary/aortic 18F-sodium fluoride uptake and pro-atherosclerosis factors in patients with multivessel coronary artery disease. *J. Nucl. Cardiol.* **2022,** *29,* 3352-3365. doi: 10.1007/s12350-022-02958-x. Epub 2022 Apr 12.
10. Raynor, W. Y.; Borja, A. J.; Zhang, V.; Kothekar, E.; Lau, H.C.; Ng, S.J.; Seraj, S.M.; Rojulpote, C.; Taghvaei, R.; Jin, K.Y.; Werner, T.J.; Høilund-Carlsen, P.F.; Alavi, A.; Revheim, M.E. Assessing Coronary Artery and Aortic Calcification in Patients with Prostate Cancer Using 18F-Sodium Fluoride PET/Computed Tomography. *PET Clin.* **2022**, *17*, 653-659. doi: 10.1016/j.cpet.2022.07.009.
11. Zhang, V.; Borja, A.J.; Rojulpote, C.; Padmanabhan, S.; Patil, S.; Gonuguntla, K.; Revheim, M.E.; Werner, T.J.; Høilund-Carlsen, P.F.; Alavi, A. Global quantification of pulmonary artery atherosclerosis using 18F-sodium fluoride PET/CT in at-risk subjects. *Am. J. Nucl. Med. Mol. Imaging.* **2020**, *10*, 119–126.
12. Gutierrez-Cardo, A.; Lillo, E.; Murcia-Casas, B.; Carrillo-Linares, J.L.; García-Argüello, F.; Sánchez-Sánchez, P.; Rodriguez-Morata, A.; Aranda, I.B.; Sánchez-Chaparro, M.Á.; García-Fernández, M.; Valdivielso, P. Skin and Arterial Wall Deposits of 18F-NaF and Severity of Disease in Patients with Pseudoxanthoma Elasticum. *J. Clin. Med.* **2020**, *9*, 1393. doi: 10.3390/jcm9051393.
13. Seraj, S.M.; Raynor, W.Y.; Revheim, M.E.; Al-Zaghal, A.; Zadeh, M.Z.; Arani, L.S.; Rojulpote, C.; Werner, T.J.; Gerke, O.; Høilund-Carlsen, P.F.; Baker, J.F.; Alavi, A.; Hunt, S.J. Assessing the feasibility of NaF-PET/CT versus FDG-PET/CT to detect abdominal aortic calcification or inflammation in rheumatoid arthritis patients. *Ann. Nucl. Med.* **2020**, *34*, 424–431. doi: 10.1007/s12149-020-01463-w.
14. Asadollahi, S.; Rojulpote, C.; Bhattaru, A.; Patil, S.; Gonuguntla, K.; Karambelkar, P.; Borja, A.J.; Vuthaluru, K.; Seraj, S.M.; Zhang, V.; Werner, T.J.; Gerke, O.; Høilund-Carlsen, P.F.; Alavi, A. Comparison of atherosclerotic burden in non-lower extremity arteries in patients with and without peripheral artery disease using 18F-NaF-PET/CT imaging. *Am. J. Nucl. Med. Mol Imaging* **2020**, *10*, 272–278.
15. Bhattaru, A.; Rojulpote, C.; Gonuguntla, K.; Patil, S.; Karambelkar, P.; Vuthaluru, K.; Zhang, V.; Borja, A.J.; Raynor, W.Y.; Werner, T.J.; Gerke, O.; Høilund-Carlsen, P.F.; Alavi, A. An understanding of the atherosclerotic molecular calcific heterogeneity between coronary, upper limb, abdominal, and lower extremity arteries as assessed by NaF PET/CT. *Am. J. Nucl. Med. Mol. Imaging* **2021**, *11*, 40–45.
16. Hayrapetian, A.; Berenji, G.R.; Nguyen, K.L.; Li, Y. 18F-Sodium fluoride uptake is associated with severity of atherosclerotic stenosis in stable ischemic heart disease. *J. Nucl. Cardiol.* **2021**, *28*, 3058–3066. doi: 10.1007/s12350-020-02238-6.
17. Ashwathanarayana, A.G.; Singhal, M.; Satapathy, S.; Sood, A.; Mittal, B.R.; Kumar, R.M.; Parmar, M.; Krishnappa, D.; Rana, N. 18F-NaF PET uptake characteristics of coronary artery culprit lesions in a cohort of patients of acute coronary syndrome with ST-elevation myocardial infarction and chronic stable angina: A hybrid fluoride PET/CTCA study. *J. Nucl. Cardiol.* **2022**, *29*, 558–568. doi: 10.1007/s12350-020-02284-0.
18. Majeed, K.; Bellinge, J.W.; Butcher, S.C.; Alcock, R.; Spiro, J.; Playford, D.; Hillis, G.S.; Newby, D.E.; Mori, T.A.; Francis, R.; Schultz, C.J. Coronary 18F-sodium fluoride PET detects high-risk plaque features on optical coherence tomography and CT-angiography in patients with acute coronary syndrome. *Atherosclerosis* **2021**, *319*, 142–148. doi: 10.1016/j.atherosclerosis.2020.12.010.
19. Mechtouff, L.; Sigovan, M.; Douek, P.; Costes, N.; Le Bars, D.; Mansuy, A.; Haesebaert, J.; Bani-Sadr, A.; Tordo, J.; Feugier, P.; Millon, A.; Luong, S.; Si-Mohamed, S.; Collet-Benzaquen, D.; Canet-Soulas, E.; Bochaton, T.; Crola Da Silva, C.; Paccalet, A.; Magne, D.; Berthezene, Y.; Nighoghossian, N. Simultaneous assessment of microcalcifications and morphological criteria of vulnerability in carotid artery plaque using hybrid 18F-NaF PET/MRI. *J. Nucl. Cardiol.* **2022**, *29*, 1064–1074. doi: 10.1007/s12350-020-02400-0.
20. Wurster, T.H.; Landmesser, U.; Abdelwahed, Y.S.; Skurk, C.; Morguet, A.; Leistner, D.M.; Fröhlich, G.; Haghikia, A.; Engel, L.C.; Schuster, A.; Noutsias, M.; Schulze, D.; Hamm, B.; Furth, C.; Brenner, W.; Botnar, R.M.; Bigalke, B.; Makowski, M.R. Simultaneous [18F]fluoride and gadobutrol enhanced coronary positron emission tomography/magnetic resonance imaging for in vivo plaque characterization. *Eur. Heart. J. Cardiovasc. Imaging* **2022**, *23*, 1391–1398. doi: 10.1093/ehjci/jeab276.

21. Kaczynski, J.; Sellers, S.; Seidman, M.A.; Syed, M.; Dennis, M.; Mcnaught, G.; Jansen, M.; Semple, S.I.; Alcaide-Corral, C.; Tavares, A.A.S.; MacGillivray, T.; Debono, S.; Forsythe, R.; Tambyraja, A.; Slomka, P.J.; Leipsic, J.; Dweck, M.R.; Whiteley, W.; Wardlaw, J.; van Beek, E.J.R.; Newby, D.E.; Williams, M.C. 18F-NaF PET/MRI for Detection of Carotid Atheroma in Acute Neurovascular Syndrome. *Radiology.* **2022**, *305*, 137-148. doi: 10.1148/radiol.212283.
22. Rojulpote, C.; Patil, S.; Gonuguntla, K.; Karambelkar, P.; Bravo, P.E.; Seraj, S.M.; Asadollahi, S.; Raynor, W.Y.; Bhattaru, A.; Borja, A.J.; Zhang, V.; Werner, T.J.; Gerke, O.; Høilund-Carlsen, P.F.; Alavi, A. NaF-PET/CT global assessment in detecting and quantifying subclinical cardiac atherosclerosis and its association with blood pressure in non-dyslipidemic individuals. *Am. J. Cardiovasc. Dis.* **2020**, *10*, 101–107.
23. Patil, S.; Rojulpote, C.; Gonuguntla, K.; Karambelkar, P.; Bhattaru, A.; Raynor, W.Y.; Borja, A.J.; Vuthaluru, K.; Zhang, V.; Werner, T.J.; Gerke, O.; Høilund-Carlsen, P.F.; Alavi, A. Association of triglyceride to high density lipoprotein ratio with global cardiac microcalcification to evaluate subclinical coronary atherosclerosis in non-diabetic individuals. *Am. J. Cardiovasc. Dis.* **2020**, *10*, 241–246.
24. Gonuguntla, K.; Rojulpote, C.; Patil, S.; Bhattaru, A.; Karambelkar, P.; Vuthaluru, K.; Raynor, W.Y.; Borja, A.J.; Zhang, V.; Werner, T.J.; Gerke, O.; Høilund-Carlsen, P.F.; Alavi, A. Utilization of NaF-PET/CT in assessing global cardiovascular calcification using CHADS2 and CHADS2-VASc scoring systems in high risk individuals for cardiovascular disease. *Am. J. Nucl. Med. Mol. Imaging* **2020**, *10*, 293–300.
25. Borja, A.J.; Bhattaru, A.; Rojulpote, C.; Hancin, E.C.; Detchou, D.K.; Patil, S.; Gonuguntla, K.; Karambelkar, P.; Chinta, S.; Vuthaluru, K.; Werner, T.J.; Gerke, O.; Høilund-Carlsen, P.F.; Alavi, A. Association between atherosclerotic cardiovascular disease risk score estimated by pooled cohort equation and coronary plaque burden as assessed by NaF-PET/CT. *Am. J. Nucl. Med. Mol. Imaging* **2020**, *10*, 312–318.
26. Paydary, K.; Revheim, M.E.; Emamzadehfard, S.; Gholami, S.; Pourhassan, S.; Werner, T.J.; Høilund-Carlsen, P.F.; Alavi, A. Quantitative thoracic aorta calcification assessment by 18F-NaF PET/CT and its correlation with atherosclerotic cardiovascular disorders and increasing age. *Eur. Radiol.* **2021**, *31*, 785–794. doi: 10.1007/s00330-020-07133-9.
27. Borges-Rosa, J.; Oliveira-Santos, M.; Silva, R.; da Silva, N.P.; Abrunhosa, A.; Castelo-Branco, M.; Gonçalves, L.; Ferreira, M.J. Cardiac microcalcification burden: Global assessment in high cardiovascular risk subjects with Na[18F]F PET-CT. *J. Nucl. Cardiol.* **2022**, *29*, 1846–1854. doi: 10.1007/s12350-021-02600-2.
28. Castro, S.A.; Muser, D.; Lee, H.; Hancin, E.C.; Borja, A.J.; Acosta, O.; Werner, T.J.; Thomassen, A.; Constantinescu, C.; Høilund-Carlsen, P.F.; Alavi, A. Carotid artery molecular calcification assessed by [18F]fluoride PET/CT: correlation with cardiovascular and thromboembolic risk factors. *Eur. Radiol.* **2021**, *31*, 8050–8059. doi: 10.1007/s00330-021-07917-7.
29. Kwiecinski, J.; Tzolos, E.; Adamson, P.D.; Cadet, S.; Moss, A.J.; Joshi, N.; Williams, M.C.; van Beek, E.J.R.; Dey, D.; Berman, D.S.; Newby, D.E.; Slomka, P.J.; Dweck, M.R. Coronary 18F-Sodium Fluoride Uptake Predicts Outcomes in Patients With Coronary Artery Disease. *J. Am. Coll. Cardiol.* **2020**, *75*, 3061–3074. doi: 10.1016/j.jacc.2020.04.046.
30. Bellinge, J.W.; Francis, R.J.; Lee, S.C.; Phillips, M.; Rajwani, A.; Lewis, J.R.; Watts, G.F.; Schultz, C.J. 18F-Sodium Fluoride Positron Emission Tomography Activity Predicts the Development of New Coronary Artery Calcifications. *Arterioscler. Thromb. Vasc. Biol.* **2021**, *41*, 534–541. doi: 10.1161/ATVBAHA.120.315364.
31. Doris, M.K.; Meah, M.N.; Moss, A.J.; Andrews, J.P.M.; Bing, R.; Gillen, R.; Weir, N.; Syed, M.; Daghem, M.; Shah, A.; Williams, M.C.; van Beek, E.J.R.; Forsyth, L.; Dey, D.; Slomka, P.J.; Dweck, M.R.; Newby, D.E.; Adamson, P.D.. Coronary 18F-Fluoride Uptake and Progression of Coronary Artery Calcification. *Circ. Cardiovasc. Imaging* **2020**, *13*, e011438. doi: 10.1161/CIRCIMAGING.120.011438.
32. Hu, Y.; Hu, P.; Hu, B.; Chen, W.; Cheng, D.; Shi, H. Dynamic monitoring of active calcification in atherosclerosis by 18F-NaF PET imaging. *Int. J. Cardiovasc. Imaging* **2021**, *37*, 731–739. doi: 10.1007/s10554-020-02019-9.
33. Fiz, F.; Piccardo, A.; Morbelli, S.; Bottoni, G.; Piana, M.; Cabria, M.; Bagnasco, M.; Sambuceti, G. Longitudinal analysis of atherosclerotic plaques evolution: an 18F-NaF PET/CT study. *J. Nucl. Cardiol.* **2022**, *29*, 1713–1723. doi: 10.1007/s12350-021-02556-3.
34. Brodsky, L.; Chesnais, H.; Piri, R.; Høilund-Carlsen, P.F.; Alavi, A.; Rajapakse, C.S. Association of baseline subject characteristics with changes in coronary calcification assessed by 18F-sodium fluoride PET/CT. *Hell. J. Nucl. Med.* **2021**, *24*, 45–52. doi: 10.1967/s002449912305.

35. Lillo, E.; Gutierrez-Cardo, A.; Murcia-Casas, B.; Carrillo-Linares, J.L.; Garcia-Argüello, F.; Chicharo de Freitas, R.; Baquero-Aranda, I.; Valdivielso, P.; García-Fernández, M.; Sánchez-Chaparro, M.Á. Cutaneous and Vascular Deposits of 18F-NaF by PET/CT in the Follow-Up of Patients with Pseudoxanthoma Elasticum. *J. Clin. Med.* **2021**, *10*, 2588. doi: 10.3390/jcm10122588.
36. Reijrink, M.; de Boer, S.A.; Te Velde-Keyzer, C.A.; Sluiter, J.K.E.; Pol, R.A.; Heerspink, H.J.L.; Greuter, M.J.W.; Hillebrands, J.L.; Mulder, D.J.; Slart, R.H.J.A. [18F]FDG and [18F]NaF as PET markers of systemic atherosclerosis progression: A longitudinal descriptive imaging study in patients with type 2 diabetes mellitus. *J. Nucl. Cardiol.* **2022**, *29*, 1702–1709. doi: 10.1007/s12350-021-02781-w.
37. Piri, R.; Lici, G.; Riyahimanesh, P.; Gerke, O.; Alavi, A.; Høilund-Carlsen, P.F. Two-year change in 18F-sodium fluoride uptake in major arteries of healthy subjects and angina pectoris patients. *Int. J. Cardiovasc. Imaging* **2021**, *37*, 3115–3126. doi: 10.1007/s10554-021-02263-7.
38. Kitagawa, T.; Sasaki, K.; Fujii, Y.; Tatsugami, F.; Awai, K.; Hirokawa, Y.; Nakano, Y. A longitudinal pilot study to assess temporal changes in coronary arterial 18F-sodium fluoride uptake. *J. Nucl. Cardiol.* **2022** Apr 29. doi: 10.1007/s12350-022-02975-w.
39. Kwiecinski, J.; Tzolos, E.; Fletcher, A.J.; Nash, J.; Meah, M.N.; Cadet, S.; Adamson, P.D.; Grodecki, K.; Joshi, N.; Williams, M.C.; van Beek, E.J.R.; Lai, C.; Tavares, A.A.S.; MacAskill, M.G.; Dey, D.; Baker, A.H.; Leipsic, J.; Berman, D.S.; Sellers, S.L.; Newby, D.E.; Dweck, M.R.; Slomka, P.J. Bypass Grafting and Native Coronary Artery Disease Activity. *JACC Cardiovasc. Imaging.* **2022**, *15*, 875-887. doi: 10.1016/j.jcmg.2021.11.030.
40. Fletcher, A.J.; Tew, Y.Y.; Tzolos, E.; Joshi, S.S.; Kaczynski, J.; Nash, J.; Debono, S.; Lembo, M.; Kwiecinski, J.; Bing, R.; Syed, M.B.J.; Doris, M.K.; van Beek, E.J.R.; Moss, A.J.; Jenkins, W.S.; Walker, N.L.; Joshi, N.V.; Pawade, T.A.; Adamson, P.D.; Whiteley, W.N.; Wardlaw, J.M.; Slomka, P.J.; Williams, M.C.; Newby, D.E.; Dweck, M.R. Thoracic Aortic 18F-Sodium Fluoride Activity and Ischemic Stroke in Patients With Established Cardiovascular Disease. *JACC Cardiovasc. Imaging.* **2022,** *15*, 1274-1288. doi: 10.1016/j.jcmg.2021.12.013. Epub 2022 Feb 16.
41. Dai, M.; Winnie Xu, W.; Chesnais, H.; Anabaraonye, N.; Parente, J.; Chatterjee, S.; Rajapakse, C.S. Atherogenic Indices as a Predictor of Aortic Calcification in Prostate Cancer Patients Assessed Using 18F-Sodium Fluoride PET/CT. *Int. J. Mol. Sci.* **2022**, *23*, 13056. doi: 10.3390/ijms232113056.
42. Hsu, J.J.; Fong, F.; Patel, R.; Qiao, R.; Lo, K.; Soundia, A.; Chang, C.C.; Le, V.; Tseng, C.H.; Demer, L.L.; Tintut, Y. Changes in microarchitecture of atherosclerotic calcification assessed by 18F-NaF PET and CT after a progressive exercise regimen in hyperlipidemic mice. *J. Nucl. Cardiol.* **2021**, *28*, 2207–2214. doi: 10.1007/s12350-019-02004-3.
43. Florea, A.; Sigl, J.P.; Morgenroth, A.; Vogg, A.; Sahnoun, S.; Winz, O.H.; Bucerius, J.; Schurgers, L.J.; Mottaghy, F.M. Sodium [18F]Fluoride PET Can Efficiently Monitor In Vivo Atherosclerotic Plaque Calcification Progression and Treatment. *Cells* **2021**, *10*, 275. doi: 10.3390/cells10020275.
44. Dietz, M.; Chironi, G.; Faraggi, M. Reduced 18F-sodium fluoride activity in coronary plaques after statin therapy. *Eur. Heart J. Cardiovasc. Imaging* **2021**, *22*, e133. doi: 10.1093/ehjci/jeab016.
45. Zhang, X.; Chen, X.; Liang, Z.; Nie, M.; Yan, Y.; Zhao, Q. Atorvastatin Promotes Macrocalcification, But Not Microcalcification in Atherosclerotic Rabbits: An 18F-NaF PET/CT Study. *J. Cardiovasc. Pharmacol.* **2021**, *78*, 544–550. doi: 10.1097/FJC.0000000000001088.
46. Bellinge, J.W.; Francis, R.J.; Lee, S.C.; Vickery, A.; Macdonald, W.; Gan, S.K.; Chew, G.T.; Phillips, M.; Lewis, J.R.; Watts, G.F.; Schultz, C.J. The effect of Vitamin-K1 and Colchicine on Vascular Calcification Activity in subjects with Diabetes Mellitus (ViKCoVaC): A double-blind 2x2 factorial randomized controlled trial. *J. Nucl. Cardiol.* **2022**, *29*, 1855–1866. doi: 10.1007/s12350-021-02589-8.
47. Bellinge, J.W.; Francis, R.J.; Lee, S.C.; Bondonno, N.P.; Sim, M.; Lewis, J.R.; Watts, G.F.; Schultz, C.J. The effect of vitamin K1 on arterial calcification activity in subjects with diabetes mellitus: a post hoc analysis of a double-blind, randomized, placebo-controlled trial. *Am. J. Clin. Nutr.* **2022**, *115*, 45–52. doi: 10.1093/ajcn/nqab306.
48. Jensen, J.K.; Binderup, T.; Grandjean, C.E.; Bentsen, S.; Ripa, R.S.; Kjaer, A. Semaglutide reduces vascular inflammation investigated by PET in a rabbit model of advanced atherosclerosis. *Atherosclerosis.* **2022**, *352*, 88-95. doi: 10.1016/j.atherosclerosis.2022.03.032.
49. Bessueille, L,; Kawtharany, L.; Quillard, T.; Goettsch, C.; Briolay, A.; Taraconat, N.; Balayssac, S.; Gilard, V.; Mebarek, S.; Peyruchaud, O.; Duboeuf, F.; Bouillot, C.; Pinkerton, A.; Mechtouff. L.; Buchet, R.; Hamade, E.; Zibara, K.; Fonta, C.; Canet-Soulas, E.; Millan, J.L.; Magne, D. Inhibition of alkaline phosphatase impairs dyslipidemia and protects mice from atherosclerosis. *Transl. Res.* **2023**, *251*, 2-13. doi: 10.1016/j.trsl.2022.06.010.

50. Irkle, A.; Vesey, A.T.; Lewis, D.Y.; Skepper, J.N.; Bird, J.L.; Dweck, M.R.; Joshi, F.R.; Gallagher, F.A.; Warburton, E.A.; Bennett, M.R.; Brindle, K.M.; Newby, D.E.; Rudd, J.H.; Davenport, A.P. Identifying active vascular microcalcification by (18)F-sodium fluoride positron emission tomography. *Nat. Commun.* **2015**, *6*, 7495. doi: 10.1038/ncomms8495.
51. Tapia-Vieyra, J.V.; Delgado-Coello, B.; Mas-Oliva, J. Atherosclerosis and Cancer; A Resemblance with Far-reaching Implications. *Arch. Med. Res.* **2017**, *48*, 12-26. doi: 10.1016/j.arcmed.2017.03.005.
52. Yuhong Diao, Y.; Liu, Z.; Chen, L.; Zhang, W.; Sun, D. The Relationship Between Cancer and Functional and Structural Markers of Subclinical Atherosclerosis: A Systematic Review and Meta-Analysis. *Front. Cardiovasc. Med.* **2022**; *9*, 849538. doi: 10.3389/fcvm.2022.849538. eCollection 2022.
53. Agca, R.; Blanken, A.B.; van Sijl, A.M.; Smulders, Y.M.; Voskuyl, A.E.; van der Laken, C.; Boellaard, R.; Nurmohamed, M.T. Arterial wall inflammation is increased in rheumatoid arthritis compared with osteoarthritis, as a marker of early atherosclerosis. *Rheumatology (Oxford)* **2021**, *60*, 3360–3368. doi: 10.1093/rheumatology/keaa789.
54. Osborne, M.T.; Abbasi, T.A.; Zureigat, H.; Tawakol, A. A vessel of progress: Aortic microcalcification activity for the quantification of 18F-NaF uptake in the thoracic aorta. *J. Nucl. Cardiol.* **2022**, *29*, 1386–1388. doi: 10.1007/s12350-021-02557-2.
55. Beheshti, M.; Mottaghy, F.M.; Paycha, F.; Behrendt, F.F.F.; Van den Wyngaert, T.; Fogelman, I.; Strobel, K.; Celli, M.; Fanti, S.; Giammarile, F.; Krause, B.; Langsteger, W. (18)F-NaF PET/CT: EANM procedure guidelines for bone imaging. *Eur. J. Nucl. Med. Mol. Imaging* **2015**, *42*, 1767–1777. doi: 10.1007/s00259-015-3138-y.
56. Segall, G.; Delbeke, D.; Stabin, M.G.; Even-Sapir, E.; Fair, J.; Sajdak, R.; Smith, G.T.; S.N.M. SNM practice guideline for sodium 18F-fluoride PET/CT bone scans 1.0. *J. Nucl. Med.* **2010**, *51*, 1813–1820. doi: 10.2967/jnumed.110.082263.
57. de Jong, E.E.C.; van Elmpt, W.; Hoekstra, O.S.; Groen, H.J.M.; Smit, E.F.; Boellaard, R.; Lambin, P.; Dingemans, A.C. Quality assessment of positron emission tomography scans: recommendations for future multicentre trials. *Acta Oncol.* **2017**, *56*, 1459–1464. doi: 10.1080/0284186X.2017.
58. Kaalep, A.; Sera, T.; Rijnsdorp, S.; Yaqub, M.; Talsma, A.; Lodge, M.A.; Boellaard, R. Feasibility of state of the art PET/CT systems performance harmonisation. *Eur. J. Nucl. Med. Mol. Imaging* **2018**, *45*, 1344–1361. doi: 10.1007/s00259-018-3977-4.
59. Blomberg, B.A.; Thomassen, A.; de Jong, P.A.; Simonsen, J.A.; Lam, M.G.; Nielsen, A.L.; Mickley, H.; Mali, W.P.; Alavi, A.; Høilund-Carlsen, P.F. Impact of Personal Characteristics and Technical Factors on Quantification of Sodium 18F-Fluoride Uptake in Human Arteries: Prospective Evaluation of Healthy Subjects. *J. Nucl. Med.* **2015**, *56*, 1534–1540. doi: 10.2967/jnumed.115.159798.
60. Alavi, A.; Werner, T.J.; Høilund-Carlsen, P.F.; Revheim, M.E. Can Target-to-Background Ratio Measurement Lead to Detection and Accurate Quantification of Atherosclerosis With FDG PET? Likely Not. *Clin. Nucl. Med.* **2022**, *47*, 532–536. doi: 10.1097/RLU.0000000000004131.
61. Blomberg, B.A.; Akers, S.R.; Saboury, B.; Mehta, N.N.; Cheng, G.; Torigian, D.A.; Lim, E.; Del Bello, C.; Werner, T.J.; Alavi, A. Delayed time-point 18F-FDG PET CT imaging enhances assessment of atherosclerotic plaque inflammation. *Nucl. Med. Commun.* **2013**, *34*, 860–867. doi: 10.1097/MNM.0b013e3283637512.
62. Bellinge, J.W.; Majeed, K.; Carr, S.S.; Jones, J.; Hong, I.; Francis, R.J.; Schultz, C.J. Coronary artery 18F-NaF PET analysis with the use of an elastic motion correction software. *J. Nucl. Cardiol.* **2020**, *27*, 952–961. doi: 10.1007/s12350-018-01587-7.
63. Kwiecinski, J.; Lassen, M.L.; Slomka, P.J. Advances in Quantitative Analysis of 18F-Sodium Fluoride Coronary Imaging. *Mol. Imaging* **2021**, *2021*, 8849429. doi: 10.1155/2021/8849429.
64. Tzolos, E.; Lassen, M.L.; Pan, T.; Kwiecinski, J.; Cadet, S.; Dey, D.; Dweck, M.R.; Newby, D.E.; Berman, D.; Slomka, P. Respiration-averaged CT versus standard CT attenuation map for correction of 18F-sodium fluoride uptake in coronary atherosclerotic lesions on hybrid PET/CT. *J. Nucl. Cardiol.* **2022**, *29*, 430–439. doi: 10.1007/s12350-020-02245-7.
65. Arbab-Zadeh, A.; Fuster, V. The myth of the "vulnerable plaque": transitioning from a focus on individual lesions to atherosclerotic disease burden for coronary artery disease risk assessment. *J. Am. Coll. Cardiol.* **2015**, *65*, 846–855. doi: 10.1016/j.jacc.2014.11.041.
66. Arbab-Zadeh, A.; Fuster, V. The Risk Continuum of Atherosclerosis and its Implications for Defining CHD by Coronary Angiography. *J. Am. Coll. Cardiol.* **2016**, *68*, 2467–2478. doi: 10.1016/j.jacc.2016.08.069.

67. McKenney-Drake, M.L.; Moghbel, M.C.; Paydary, K.; Alloosh, M.; Houshmand, S.; Moe, S.; Salavati, A.; Sturek, J.M.; Territo, P.R.; Weaver, C.; Werner, T.J.; Høilund-Carlsen, P.F.; Sturek, M.; Alavi, A. 18F-NaF and 18F-FDG as molecular probes in the evaluation of atherosclerosis. *Eur. J. Nucl. Med. Mol. Imaging* **2018**, *45*, 2190–2200. doi: 10.1007/s00259-018-4078-0.
68. Minderhoud, S.C.S.; Fletcher, A.J.; MacNaught, G.; Cadet, S.; Korteland, S.A.; Kardys, I.; Rizopoulos, D.; Slomka, P.; Newby, D.E.; Roos-Hesselink, J.W.; Walker, N.L.; Semple, S.; Hirsch, A.; Dweck, M.R.; Wentzel, J.J. Vascular biomechanics and molecular disease activity in the thoracic aorta: a novel imaging method. *Eur. Heart. J. Cardiovasc Imaging*. **2022,** *23*, 1698-1707. doi: 10.1093/ehjci/jeac090.
69. Fletcher, A.J.; Lembo, M.; Kwiecinski, J.; Syed, M.B.J.; Nash, J.; Tzolos, E.; Bing, R.; Cadet, S.; MacNaught, G.; van Beek, E.J.R.; Moss, A.J.; Doris, M.K.; Walker, N.L.; Dey, D.; Adamson, P.D.; Newby, D.E.; Slomka, P.J.; Dweck, M.R. Quantifying microcalcification activity in the thoracic aorta. *J. Nucl. Cardiol.* **2022**, *29*, 1372–1385. doi: 10.1007/s12350-020-02458-w.
70. Ng, A.C.T.; van Rosendael, A.R.; Bax, J.J. Automated artificial intelligence quantification of aortic atherosclerotic calcifications by 18F-sodium fluoride PET/CT. *J. Nucl. Cardiol.* **2022**, *29*, 2011–2012. doi: 10.1007/s12350-021-02700-z.
71. Piri, R.; Edenbrandt, L.; Larsson, M.; Enqvist, O.; Nøddeskou-Fink, A.H.; Gerke, O.; Høilund-Carlsen, P.F. Aortic wall segmentation in 18F-sodium fluoride PET/CT scans: Head-to-head comparison of artificial intelligence-based versus manual segmentation. *J. Nucl. Cardiol.* **2022**, *29*, 2001–2010. doi: 10.1007/s12350-021-02649-z.
72. Piri, R.; Edenbrandt, L.; Larsson, M.; Enqvist, O.; Skovrup, S.; Iversen, K.K.; Saboury, B.; Alavi, A.; Gerke, O.; Høilund-Carlsen, P.F. "Global" cardiac atherosclerotic burden assessed by artificial intelligence-based versus manual segmentation in 18F-sodium fluoride PET/CT scans: Head-to-head comparison. *J. Nucl. Cardiol.* **2022**, *29*, 2531–2539. doi: 10.1007/s12350-021-02758-9.
73. Saboury, B.; Edenbrandt, L.; Piri, R.; Gerke, O.; Werner, T.; Arbab-Zadeh, A.; Alavi, A.; Høilund-Carlsen, P.F. Alavi-Carlsen Calcification Score (ACCS): A Simple Measure of Global Cardiac Atherosclerosis Burden. *Diagnostics (Basel)* **2021**, *11*, 1421. doi: 10.3390/diagnostics11081421.
74. Kwiecinski, J.; Tzolos, E.; Meah, M.N.; Cadet, S.; Adamson, P.D.; Grodecki, K.; Joshi, N.V.; Moss, A.J.; Williams, M.C.; van Beek, E.J.R.; Berman, D.S.; Newby, D.E.; Dey, D.; Dweck, M.R.; Slomka, P.J. Machine Learning with 18F-Sodium Fluoride PET and Quantitative Plaque Analysis on CT Angiography for the Future Risk of Myocardial Infarction. *J. Nucl. Med.* **2022**, *63*, 158–165. doi: 10.2967/jnumed.121.262283.
75. Sturek, M.; Alloosh, M.; Sellke, F.W. Swine Disease Models for Optimal Vascular Engineering. *Annu. Rev. Biomed. Eng.* **2020**, *22*, 25–49. doi: 10.1146/annurev-bioeng-082919-053009.
76. Gerke, O.; Ehlers, K.; Motschall, E.; Høilund-Carlsen, P.F.; Vach, W. PET/CT-Based Response Evaluation in Cancer-a Systematic Review of Design Issues. *Mol. Imaging Biol.* **2020**, *22*, 33–46. doi: 10.1007/s11307-019-01351-4.
